# Physiological and biochemical pathway: understanding their influence on maize grain yield through genotype–trait interactions

**DOI:** 10.1038/s41598-025-02628-1

**Published:** 2025-05-26

**Authors:** Pooria Mazloom, Seyed Habib Shojaie, Seyed Mohammad Nasir Mousavi

**Affiliations:** 1 Department of Agronomy, Chalous Branch, Islamic Azad University, Chalous, Iran; 2 Department of Biotechnology and Plant Breeding, Faculty of Agriculture and Food Science and Technology, Science and Research Branch, Islamic Azad University, Tehran, 1477893855 Iran; 3 Department of Animal Science and Aquaculture, Dalhousie University, Truro, NS Canada; 4 Institute of Land Use, Engineering and Precision Farming Technology, University of Debrecen, Debrecen, Hungary

**Keywords:** Maize, Graphical analysis, Correlation, Genotype × trait (GT), Polygon view, Plant ecology, Plant stress responses

## Abstract

An experiment using a randomized complete block design (RCBD) with three replications was conducted on 10 corn genotypes to evaluate the effect of genotype × trait interaction on grain yield. Analysis of variance revealed that all genotypes differed significantly (*p* < 0.01) across all traits. Duncan’s multiple range test identified KSC704 and KSC706 as favorable genotypes, while SC540 and KSC260 were considered unfavorable. Correlation analysis showed a positive association between grain yield and chlorophyll a, chlorophyll b, phosphorus, carotenoids, sodium, catalase, and potassium. Principal component analysis indicated that the first five components accounted for over 79% of the total data variance. Based on the first two principal components, the genotypes were grouped into four distinct clusters, and the traits into three. A graphical assessment of genotype performance was also conducted. According to the polygon view, genotypes KSC705, KSC400, KSC706, DC370, SC540, and KSC260 were identified as favorable. Among them, KSC705 and KSC400 were selected as the most desirable genotypes based on trait-based and ideal-genotype ranking diagrams. The biplot analysis further confirmed the grouping of genotypes into four distinct clusters.

## Introduction

Crop plants are among the most important sources of food, with cereals being a major group within this category. Among cereals, maize (*Zea mays* L.) ranks second globally in terms of cultivated area, following wheat^1^. Breeders aim to identify superior individuals to enhance economic yield and improve related physiological traits. Evaluating individuals based on agronomic characteristics—such as grain yield and its components—is a practical and efficient method for improving yield potential. Physiological traits, in particular, play a crucial role in identifying high-yielding cultivars^2^. To boost seed production, breeders focus on understanding the relationships between grain yield and other traits^3^.

Chlorophyll content is one of the key physiological traits influencing photosynthesis, which directly affects biomass accumulation and yield^4^. Multivariate statistical methods offer the ability to analyze multiple traits simultaneously, providing a comprehensive assessment of genetic diversity. Principal component analysis (PCA) is one such approach that allows the decomposition and interpretation of variation among traits^5^. Furthermore, Genotype × Trait (GT) biplot analysis is a powerful graphical method for exploring relationships among traits and identifying superior genotypes^6,7^. In a GT biplot, genotypes are represented as lines and traits as testers, facilitating the visualization of correlations and enabling indirect selection for improved grain yield^8,9^. Two experiments were conducted on corn hybrids using the GT biplot method, and the results concluded that graphical analysis and the GT biplot method are essential for trait analysis and for examining the relationships between genotypes and traits^10^.

Previous studies, such as that by Moharramnejad and Shiri^4^, have employed correlation and cluster analyses to explore genetic diversity among maize genotypes, highlighting the utility of chlorophyll fluorescence in selecting high-performing individuals. Similarly, Adedeji et al.^11^ reported significant positive correlations between various traits and grain yield in their genotype–trait correlation study.

This research aims to:

1. Compare different genotypes based on physiological traits and identify the most desirable ones.
2. Investigate the effects of genotype on various traits.
3. Examine the strength and nature of relationships between traits, with a focus on correlations between physiological traits and seed production.
4. Decompose the studied traits into principal components.
5. Utilize graphical methods to evaluate genotypes and traits within the experimental framework.

## Materials and methods

An experiment was conducted using a randomized complete block design (RCBD) with three replications on 10 corn (*Zea mays* L.) genotypes to evaluate the genotype × trait (GT) interaction and explore the relationships among various biochemical and physiological traits, as well as their influence on grain yield. The evaluated genotypes included SC540 (G1), KSC260 (G2), KSC705 (G3), SC307 (G4), KSC400 (G5), KSC700 (G6), KSC706 (G7), DC370 (G8), KSC704 (G9), and SC500 (G10). A total of ten traits were assessed: nine physiological traits—proline (PRO), catalase (CAT), grain phosphorus (GP), chlorophyll a (CHA), chlorophyll b (CHB), carotenoids (CAR), grain protein (GPR), sodium (SOD), and potassium (POT)—as well as grain yield.

The experiment was carried out in the Varamin region (51°38′49.9128″E, 35°19′30.8676″N), which receives an average annual rainfall of 218 mm. Planting, growth management, and harvesting of the genotypes were conducted with precision. Each plot consisted of four rows, each 2 m long with 75 cm spacing between rows. Samples for data collection were taken from the two central rows to minimize edge effects.

### Trait measurement methods

- **Proline Content:** One gram of fresh leaf tissue was homogenized in 10 ml of 3% sulfosalicylic acid, centrifuged at 3000 rpm for 10 min at 4°C. To 2 ml of the supernatant, 2 ml of ninhydrin reagent (prepared with 1.25 g of ninhydrin in glacial acetic acid) and 2 ml of 6 M phosphoric acid were added, followed by 2 ml of pure glacial acetic acid. Samples were heated in a water bath for 1 h, then mixed with 4 ml of toluene and vortexed for 15–20 s. Absorbance of the colored upper phase was measured at 520 nm using a UV 2100 spectrophotometer (Unico, USA), and proline concentration was calculated using a standard curve.
- **Chlorophyll and Carotenoids:** Measured following Arnon’s method. A 0.1 g leaf sample was ground in 3 ml of 80% acetone, and the volume adjusted to 15 ml. After centrifugation at 5000 rpm for 10 min, absorbance was recorded at 480, 510, 645, and 663 nm using a Shimadzu UV-160 spectrophotometer. Chlorophyll a, chlorophyll b, total chlorophyll, and carotenoid content were calculated as follows:
  - Chlorophyll a (mg/g) = [12.7 × A663 − 2.69 × A645] × V/(1000 × W)
  - Chlorophyll b (mg/g) = [22.9 × A645 − 4.68 × A663] × V/(1000 × W)
  - Total chlorophyll (mg/g) = [20.2 × A645 + 8.02 × A663] × V/(1000 × W)
  - Carotenoids (mg/g) = [A480 + (0.114 × A663) − (0.638 × A645)] × V/(1000 × W)
- **Sodium and Potassium:** These were quantified using dry ashing. One gram of dried, ground plant tissue was incinerated in an electric furnace at 550°C for 6 h. The ash was treated with 5 ml of 1 N HCl in a water bath at 80°C for 15 min, filtered, and diluted to 100 ml with distilled water. Sodium and potassium concentrations were measured using a JENWAY PEP7 flame photometer, calibrated with appropriate standards.
- **Phosphorus and Molybdenum:** Phosphorus was analyzed using a JENWAY spectrophotometer, while molybdenum was measured using a Thermo Elemental Solar S Series atomic absorption spectrometer.
- **Catalase Activity:** Assessed using the Chance and Maehly method. The reaction mixture (3 ml) consisted of 15 mM H₂O₂, 50 mM phosphate buffer (pH 7.0), and 100 µL enzyme extract. The decrease in H₂O₂ absorbance at 240 nm was measured over one minute. One unit of catalase activity was defined as the amount of enzyme decomposing 1 µmol of H₂O₂ per minute.

### Statistical analysis

Analysis of variance and mean comparisons were performed using Duncan’s multiple range test via SAS v9. Correlation analyses, including biplots and correlation maps, were used to examine the strength and direction of relationships among traits. Principal component analysis (PCA) was used to decompose trait variation and classify genotypes based on the first two principal components. Multivariate graphs, genotype rankings by trait, rankings by ideal genotype, and genotype groupings were produced using XLSTAT 2020 and GenStat v12.2.

## Results

### Analysis of variance and mean comparison

Analysis of variance (ANOVA) revealed that the genotypes differed significantly (*p* ≤ 0.01) for all examined traits, indicating substantial genetic variability among them. While the physiological traits were not significantly influenced by block effects, grain yield showed a significant block effect. The coefficient of variation (CV) ranged from 11.03% to 13.04%, with the highest CV observed for proline content and the lowest for potassium content (Table 1). Based on the mean comparison using Duncan’s multiple range test, genotypes were ranked for each trait. Among all genotypes, KSC704 and KSC706 consistently demonstrated superior performance across the majority of traits and were classified as desirable. In contrast, SC540 and KSC260 exhibited the lowest mean values for most traits and were identified as the least desirable genotypes. The overall ranking of genotypes from most to least desirable is presented in Table 2.

$$\begin{aligned} & {\text{KSC7}}0{4} > {\text{ KSC7}}0{6} > {\text{ KSC7}}0{5} > {\text{ KSC7}}00 > {\text{ KSC4}}00 > \\ & \quad \quad {\text{SC3}}0{7} > {\text{ SC5}}00 > {\text{ DC37}}0 > {\text{ KSC26}}0 > {\text{SC54}}0. \\ \end{aligned}$$

**Table 1:** Variance analysis of the 10 studied genotypes in terms of traits evaluated in the experiment.

S.OV	Df	PRO	CAT	GP	CHA	CHB	CAR	GPR	SOD	POT	GYD
Genotype	9	10,726.9*	5.07*	1.73*	4.39*	0.16*	0.006*	6.93**	18.23*	207.1*	4,982,383.1*
Block	12	1829.08^ns^	0.85^ns^	0.04^ns^	3.56^ns^	0.1^ns^	0.001^ns^	0.88^ns^	57.7^ns^	67.9^ns^	662,879.7*
Error	8	13,629	2.39	1.12	3.17	0.18	0.009	5.33	34.07	285.2	1,703,003.4
CV%	–	30.4	30.3	24.5	15.4	13.4	15.04	27.5	18.7	11.03	19.27

Differed significantly from each other at the 0.01 probability level and ns not significant.

**Table 2:** Mean comparison by Duncan’s method on the traits evaluated in the experiment.

Genotypes	Rank	PRO	CAT	GP	CHA	CHB	CAR	GPR	SOD	POT	GYD
SC540	10	361.7bc	2.91c	1.8e	11.07bc	2.6c	0.63abc	9.2abc	30abc	163.3ab	5555bc
KSC260	9	413.1ab	4.61abc	2.6de	10.4bcd	3.2abc	0.72a	8.6abc	32.3abc	150bc	6035bc
KSC705	3	337.3bcde	4.16bc	4.7a	10.97bcd	3.33a	0.72a	7.6bcde	29cde	152.6abc	8733a
SC307	6	457.4a	4.36bc	3.6ab	9.8bcde	3.3ab	0.63abc	8.2bcd	29.6bcd	158abc	6045bc
KSC400	5	262.2e	6.57a	2.7cde	10.4bcd	3.35a	0.72a	8.3bc	32.3abc	142.6cd	7338abc
KSC700	4	292.02cde	5.76ab	2.7cde	11.8abc	3.3ab	0.63abc	7.8bc	33.3ab	153.3abc	7556ab
KSC706	2	357.4bcd	6.71a	3.3bc	13.3a	3.2abc	0.6c	5.84de	35.3a	146.3bcd	7523abc
DC370	8	315.7bcde	5.89ab	2.9cd	13.3a	3.05bc	0.63abc	7.04bcde	26.6de	158abc	7799ab
KSC704	1	391.7abc	6.2a	2.7cde	12.01ab	3.2abc	0.66ab	10.05ab	32.3abc	165.6a	7342abc
SC500	7	406.8ab	3.78bc	3.2bc	12.4ab	3.34a	0.62bc	11.2a	31abc	141d	4285c

Means followed by the same letter in the column do not differ from each other, at 1% or 5% probability, according to Duncan’s test.

PRO: Proline, CAT: Catalase, GP: Grain Phosphor, CHA: Chlorophyll a, CHB: Chlorophyll b, CAR: Carotenoid, GPR: Grain Protein, SOD: Sodium, POT: Potassium, GYD: Grain Yield).

### Correlation between traits

A correlation chart was employed to assess the strength and direction of relationships among the traits studied. In the biplot diagram, the cosine of the angle between trait vectors represents the degree of correlation: acute angles (< 90°) indicate a positive correlation, right angles (= 90°) suggest no correlation, obtuse angles (> 90°) reflect a negative correlation, and an angle of 180° indicates a strong negative correlation^12^. The biplot analysis revealed a positive correlation between grain yield and several physiological traits, including chlorophyll b, seed phosphorus, carotenoids, sodium, catalase, potassium, and chlorophyll a. Additionally, a positive correlation was observed between proline and seed protein content, as well as between chlorophyll a, sodium, and catalase.

**Fig. 1:**
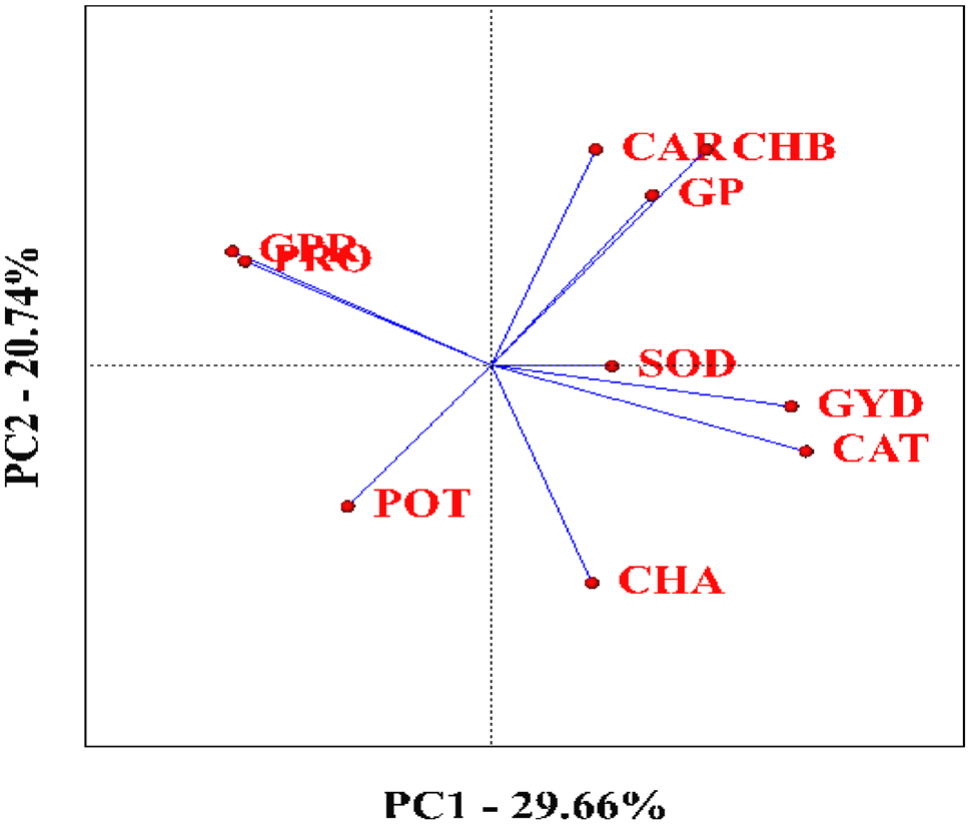
Correlation diagram between the traits evaluated in the experiment. PRO: Proline, CAT: Catalase, GP: Grain Phosphor, CHA: Chlorophyll a, CHB: Chlorophyll b, CAR: Carotenoid, GPR: Grain Protein, SOD: Sodium, POT: Potassium, GYD: Grain Yield.

Conversely, a strong negative correlation was observed between catalase and both proline and seed protein, as indicated by the 180° angle between their vectors. Negative correlations were also found between seed protein and chlorophyll b, as well as between potassium and both chlorophyll a and b. A 90° angle between catalase and potassium vectors signified no correlation between these two traits (Fig. 1).

**Fig. 2:**
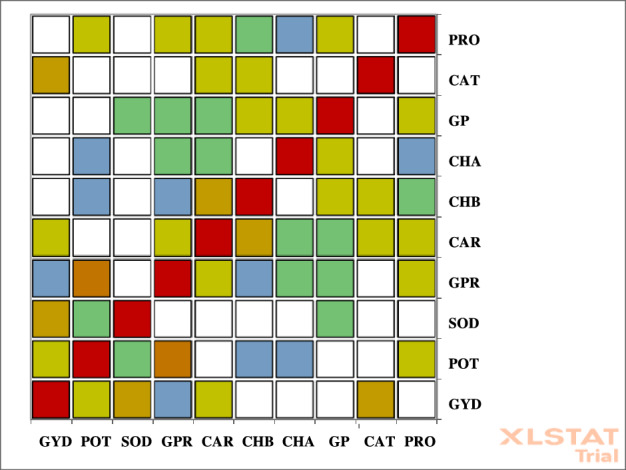
The intensity of correlation between the traits evaluated in the experiment. PRO: Proline, CAT: Catalase, GP: Grain Phosphor, CHA: Chlorophyll a, CHB: Chlorophyll b, CAR: Carotenoid, GPR: Grain Protein, SOD: Sodium, POT: Potassium, GYD: Grain Yield.

Color-coded correlation diagrams provided further insight: red signified strong positive correlations, blue represented strong negative correlations, and white indicated no correlation (Fig. 2). The highest correlation intensities were observed between potassium and phosphorus, grain yield and both catalase and sodium, and carotenoid and chlorophyll b. The weakest correlations were between chlorophyll a and proline, potassium with both chlorophyll a and b, seed protein with chlorophyll b, and grain yield with seed protein. Traits associated with white areas in the chart were interpreted as having zero correlation.

**Fig. 3:**
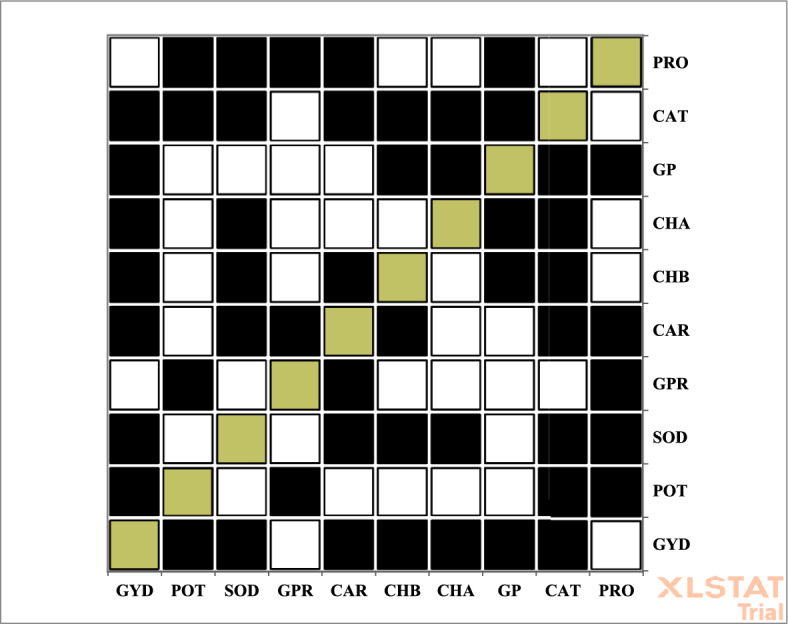
Display of + / − correlation coefficients between traits studied in the experiment. PRO: Proline, CAT: Catalase, GP: Grain Phosphor, CHA: Chlorophyll a, CHB: Chlorophyll b, CAR: Carotenoid, GPR: Grain Protein, SOD: Sodium, POT: Potassium, GYD: Grain Yield.

Figure 3 presented positive and negative correlation coefficients in a binary color scheme: black for positive and white for negative correlations. Notably, seed protein showed significant correlations with proline and catalase; chlorophyll a and b correlated with seed protein; carotenoids were associated with proline and catalase; and sodium and potassium were also correlated with proline and catalase. Grain yield showed positive correlations with catalase, seed protein, chlorophyll a, chlorophyll b, carotenoids, sodium, and potassium. A detailed summary of these correlation coefficients is provided in Table 3.

**Table 3:** +/ − Correlation coefficients between traits studied in the experiment.

Traits	Correlation coefficients +	Correlation coefficients −
Catalase (CAT)	–	PRO
Grain Phosphor (GP)	PRO,CAT	–
Chlorophyll a (CHA)	CAT,GP	PRO
Chlorophyll b (CHB)	CAT,GP	PRO,CHA
Carotenoid (CAR)	PRO,CAT,CHB	GP,CHA
Grain Protein (GPR)	PRO,CAR	CAT,GP,CHA,CHB
Sodium (SOD)	PRO,CAT,CHA,CHB,CAR	GP,GPR
Potassium (POT)	PRO,CAT,GPR	GP,CHA,CHB,CAR,SOD
Grain Yield (GYD)	CAT,GP,CHA,CHB,CAR,SOD,POT	PRO,GPR

### Principal components analysis (PCA)

**Fig. 4:**
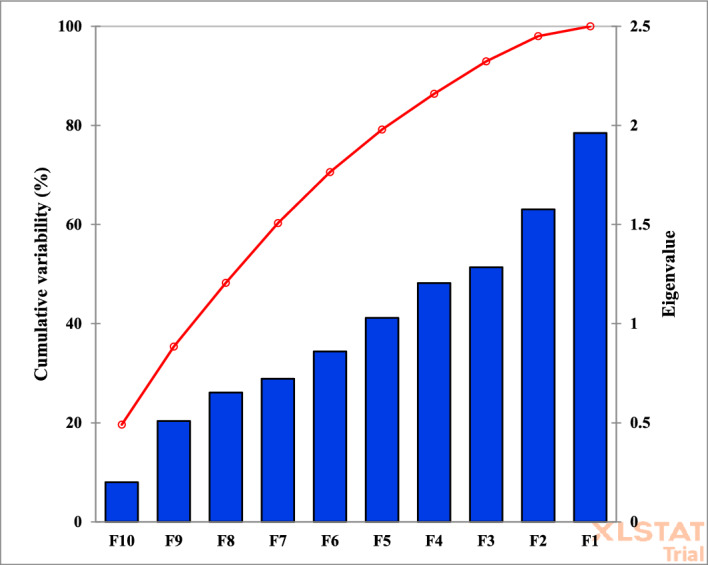
Vector eigenvalue diagram.

As one of the primary objectives of this study, principal component analysis (PCA) was employed to describe genetic diversity among genotypes, assess the contribution of each trait to overall variability, and reduce data dimensionality by identifying uncorrelated components—linear combinations of the original variables^13^. Based on the eigenvalue scree plot (Fig. 4), the first five principal components collectively accounted for the majority of variance in the dataset. Specifically, the first principal component (PC1) explained 19% of the total variance, followed by PC2 (15%), PC3 (12%), PC4 (10%), and PC5 (8%), cumulatively capturing over 79% of the total variation.

In PC1, traits such as chlorophyll a (0.33), chlorophyll b (0.37), catalase, phosphorus, sodium, and grain yield contributed positively, indicating their strong influence on this component. PC2 was primarily driven by carotenoids (0.49) and catalase (0.41), along with positive contributions from proline, chlorophyll b, and other related traits. In PC3, grain yield had the most substantial positive loading (0.48), followed by catalase, phosphorus, chlorophyll a, and potassium, all of which exhibited meaningful contributions. The fourth component (PC4) was dominated by seed phosphorus (0.71), while other traits including proline, catalase, chlorophyll b, carotenoid, potassium, and grain yield also showed positive effects. PC5 was strongly influenced by sodium (0.62), alongside contributions from proline, phosphorus, chlorophyll a, and grain yield (Table 4).

**Table 4:** Principal components analysis on the traits studied in the experiment.

	PC1	PC2	PC3	PC4	PC5	PC6	PC7	PC8	PC9	PC10
PRO	− 0.29	0.21	− 0.12	0.4	0.58	0.13	− 0.34	− 0.08	0.39	0.22
CAT	0.2	0.41	0.31	0.07	− 0.31	0.15	− 0.66	0.3	− 0.08	− 0.13
GP	0.16	− 0.14	0.2	0.71	0.18	0.23	0.27	0.14	− 0.41	− 0.21
CHA	0.33	− 0.21	0.3	− 0.21	0.04	0.69	0.13	− 0.06	0.43	0.05
CHB	0.37	0.2	− 0.43	0.23	− 0.21	− 0.08	0.28	0.5	0.33	0.26
CAR	− 0.003	0.49	− 0.45	0.03	− 0.17	0.39	0.14	− 0.45	− 0.09	− 0.34
GPR	− 0.54	0.09	− 0.006	− 0.11	− 0.17	0.44	0.13	0.3	− 0.23	0.47
SOD	0.13	0.32	− 0.03	− 0.44	0.62	0.02	0.14	0.39	− 0.2	− 0.25
POT	− 0.46	0.23	0.38	0.08	− 0.16	− 0.13	0.35	0.18	0.43	− 0.42
GYD	0.22	0.5	0.45	0.02	0.05	− 0.21	0.28	− 0.35	− 0.09	0.47
Proportion	0.19	0.15	0.12	0.12	0.1	0.08	0.07	0.06	0.05	0.02
Cumulative	0.19	0.35	0.48	0.6	0.7	0.79	0.86	0.92	0.98	1

PRO: Proline, CAT: Catalase, GP: Grain Phosphor, CHA: Chlorophyll a, CHB: Chlorophyll b, CAR: Carotenoid, GPR: Grain Protein, SOD: Sodium, POT: Potassium, GYD: Grain Yield.

**Fig. 5:**
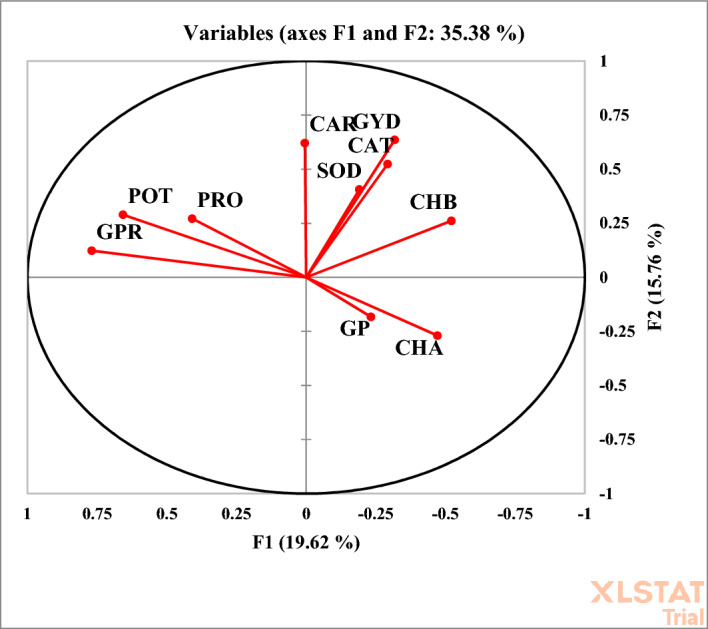
The grouping of evaluated traits based on the first and second main components. PRO: Proline, CAT: Catalase, GP: Grain Phosphor, CHA: Chlorophyll a, CHB: Chlorophyll b, CAR: Carotenoid, GPR: Grain Protein, SOD: Sodium, POT: Potassium, GYD: Grain Yield.

**Fig. 6:**
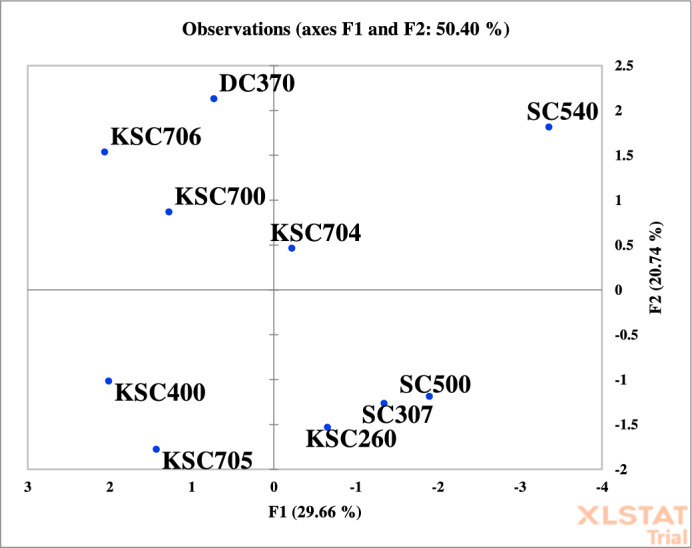
Grouping diagram of genotypes based on the first and second principal components.

Trait clustering based on the first two principal components allowed for classification into three distinct groups (Fig. 5). Group I comprised carotenoids, grain yield, catalase, sodium, and chlorophyll b. Group II included chlorophyll a and seed phosphorus, while Group III was composed of proline, potassium, and seed protein. Similarly, genotypes were grouped based on their scores along the first two principal components (Fig. 6). SC540 and KSC704 were assigned to Group I; SC500, SC307, and KSC260 to Group II; KSC705 and KSC400 to Group III; and KSC700, KSC706, and DC370 to Group IV. This multivariate analysis enabled the identification of high-performing and desirable genotypes based on their physiological trait profiles.

### Polygon diagram

A graphical analysis approach was employed to evaluate the relationship between genotypes and grain yield, as well as its associated components. A polygon (GGE biplot) diagram was used to identify the most desirable genotypes based on their multivariate performance. This method involves connecting the genotypes farthest from the origin to form a polygon, which visually partitions the genotypes into sectors based on performance. The first and second principal components explained 26.66% and 20.74% of the total variance, respectively, accounting for a combined 50.4% of the observed variation.

Based on this analysis, genotypes KSC705, KSC400, KSC706, DC370, SC540, SC500, and KSC260 were positioned furthest from the origin and thus identified as high-performing and desirable genotypes. Specifically, KSC705 exhibited superiority in traits such as chlorophyll b, carotenoids, and seed phosphorus. The DC370 genotype excelled in chlorophyll a content, while SC500 and SC307 were favorable in terms of seed protein and proline accumulation. These findings highlight the genotypic potential for targeted physiological and biochemical trait enhancement.

**Fig. 7:**
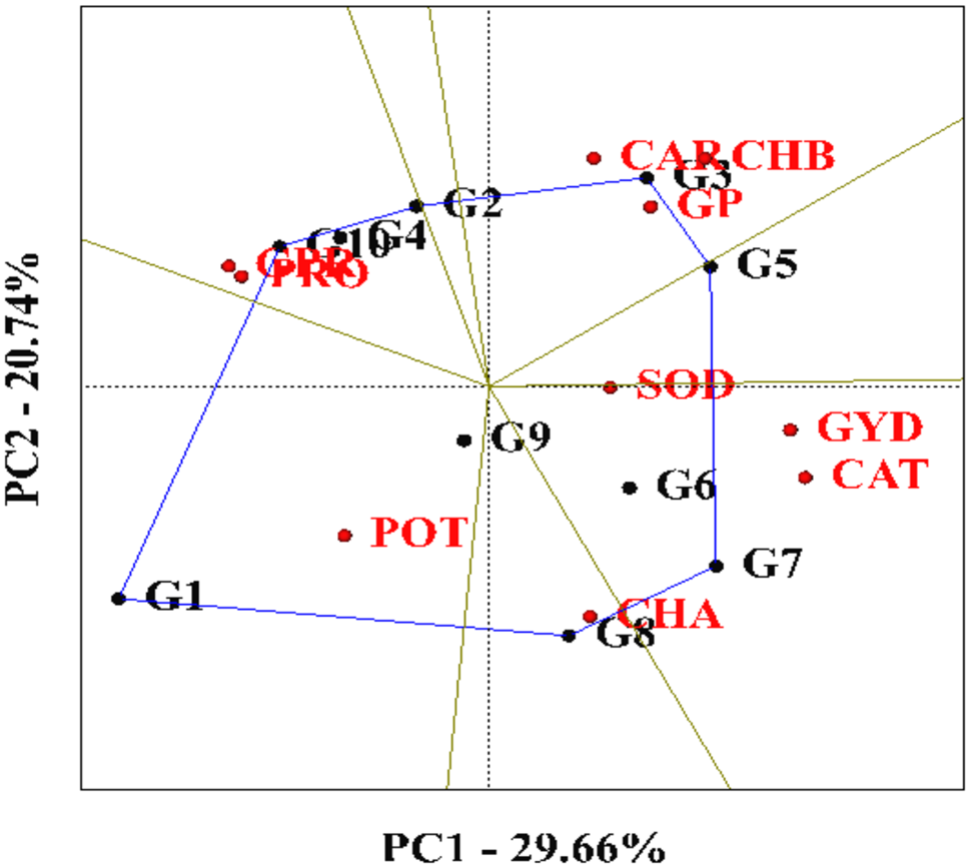
Polygon biplot of genotypes examined in the experiment based on the evaluated traits. G1: SC540, G2: KSC260, G3: KSC705, G4: SC307, G5: KSC400, G6: KSC700, G7: KSC706, G8: DC370, G9: KSC704, G10: SC500. PRO: Proline, CAT: Catalase, GP: Grain Phosphor, CHA: Chlorophyll a, CHB: Chlorophyll b, CAR: Carotenoid, GPR: Grain Protein, SOD: Sodium, POT: Potassium, GYD: Grain Yield.

In contrast, the KSC704 genotype was located close to the origin of the polygon diagram, indicating a neutral response to changes in the measured traits and suggesting limited phenotypic divergence. A similar graphical analysis methodology was previously applied by Karaman et al.^8^ to evaluate genotype performance in their study (Fig. 7).

### Ranking of genotypes in terms of stability in traits

**Fig. 8:**
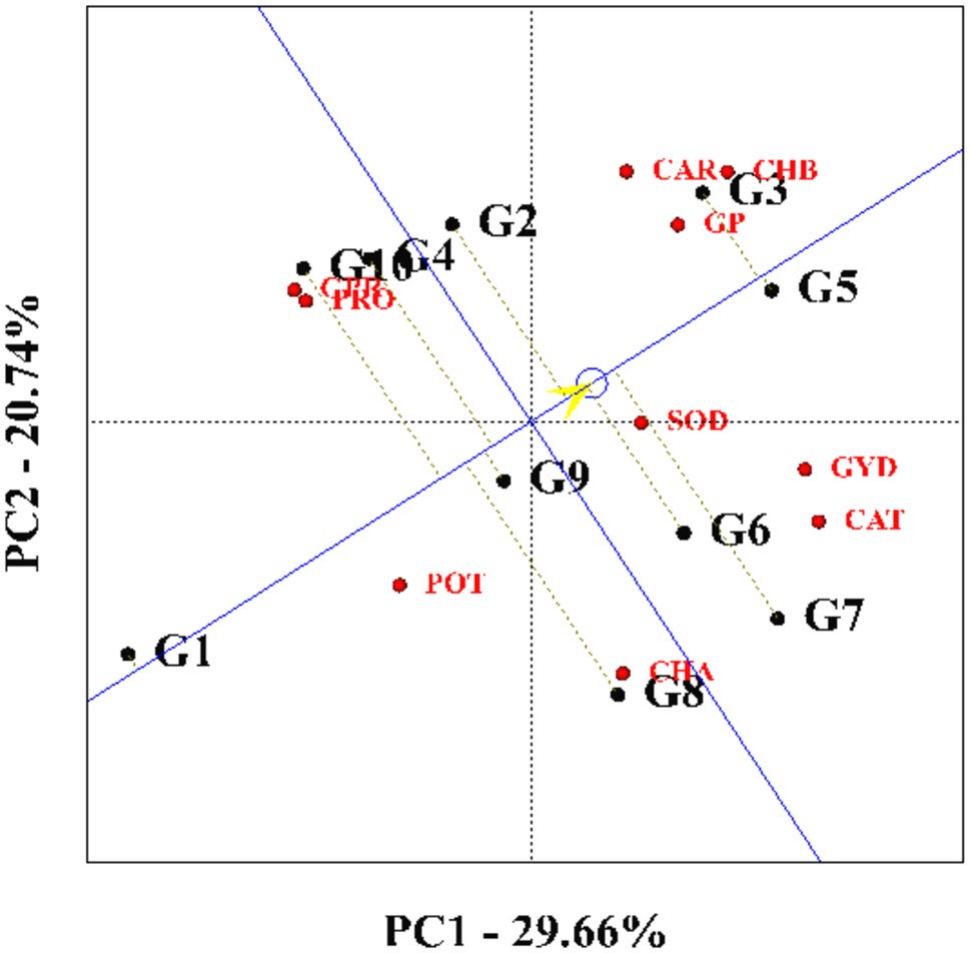
Biplot ranking of genotypes in terms of the stability of traits evaluated in the experiment. G1: SC540, G2: KSC260, G3: KSC705, G4: SC307, G5: KSC400, G6: KSC700, G7: KSC706, G8: DC370, G9: KSC704, G10: SC500. PRO: Proline, CAT: Catalase, GP: Grain Phosphor, CHA: Chlorophyll a, CHB: Chlorophyll b, CAR: Carotenoid, GPR: Grain Protein, SOD: Sodium, POT: Potassium, GYD: Grain Yield.

In this graph, which is connected from the origin of the linear coordinates to the average of the traits, and those figures located at the positive beginning of this axis have higher performance and desirable traits, while those figures near this axis exhibit a higher degree of balance in terms of the examined characteristics. Based on the ranking chart of genotypes in terms of their stability against traits, the KSC705 genotype was identified as the most desirable genotype in terms of all traits, and the SC540 genotype was identified as the least desirable genotype. Due to their proximity to the mean axis, three genotypes, KSC400, KSC705, and KSC704, were also identified as stable genotypes. Following is a ranking of genotypes from most desirable to least desirable: (Fig. 8).

$$\begin{aligned} & {\text{KSC7}}0{5} > {\text{ KSC4}}00 > {\text{ KSC7}}0{6} > {\text{ KSC7}}00 > {\text{ KSC26}}0 > \\ & \quad \quad {\text{SC3}}0{7} > {\text{ KSC7}}0{4} > {\text{ DC37}}0 > {\text{ SC5}}00 > {\text{ SC54}}0. \\ \end{aligned}$$

### Ranking of genotypes based on the ideal genotype

**Fig. 9:**
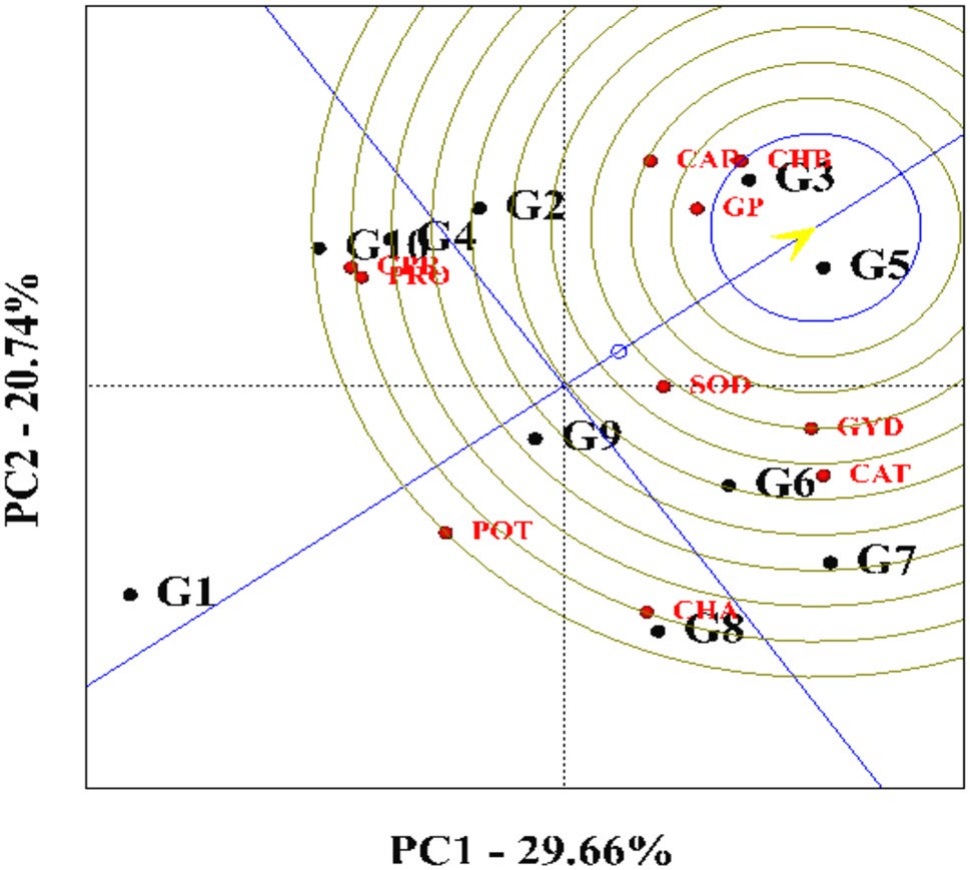
Ranking of genotypes based on the ideal genotype. G1: SC540, G2: KSC260, G3: KSC705, G4: SC307, G5: KSC400, G6: KSC700, G7: KSC706, G8: DC370, G9: KSC704, G10: SC500. PRO: Proline, CAT: Catalase, GP: Grain Phosphor, CHA: Chlorophyll a, CHB: Chlorophyll b, CAR: Carotenoid, GPR: Grain Protein, SOD: Sodium, POT: Potassium, GYD: Grain Yield.

To select the best genotype, a ranking diagram was drawn based on the ideal genotype (Fig. 9). An arrow indicates that the best point on this diagram is the center of the concentric circles, and other genotypes are ranked based on this point. A hypothetical ideal genotype is defined based on the most stable and productive genotype^12^. In accordance with the ranking chart of genotypes based on ideal genotype, KSC400 and KSC705 genotypes were identified as favorable genotypes and SC540 genotype as unfavorable genotypes. Following is a ranking of genotypes from the most desirable to the least desirable:

$$\begin{aligned} & {\text{KSC7}}0{5} > {\text{ KSC4}}00 > {\text{ KSC7}}00 > {\text{ KSC26}}0 > {\text{ KSC7}}0{6} > \\ & \quad \quad {\text{KSC7}}0{4} > {\text{ SC3}}0{7} > {\text{ DC37}}0 > {\text{ SC5}}00 > {\text{SC54}}0. \\ \end{aligned}$$

### Grouping of genotypes

**Fig. 10:**
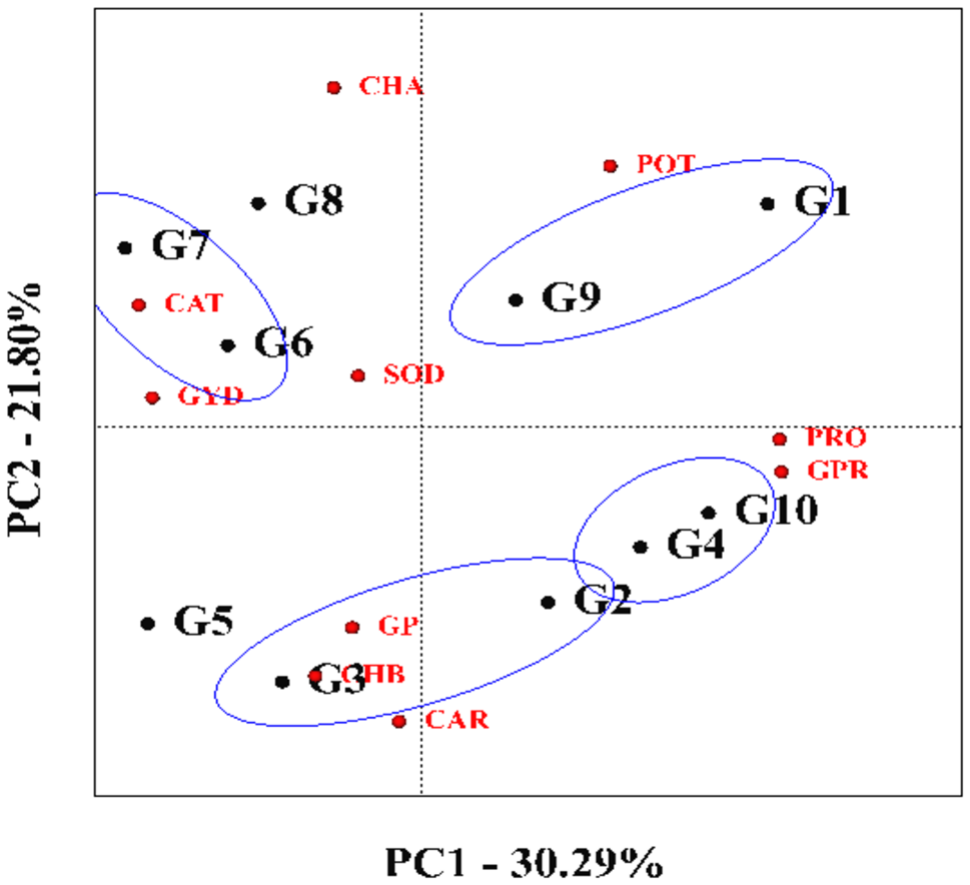
Biplot of the grouping of genotypes in terms of traits evaluated in the experiment. G1: SC540, G2: KSC260, G3: KSC705, G4: SC307, G5: KSC400, G6: KSC700, G7: KSC706, G8: DC370, G9: KSC704, G10: SC500. PRO: Proline, CAT: Catalase, GP: Grain Phosphor, CHA: Chlorophyll a, CHB: Chlorophyll b, CAR: Carotenoid, GPR: Grain Protein, SOD: Sodium, POT: Potassium, GYD: Grain Yield.

The genotype grouping chart evaluates hybrids based on their stability and performance in various traits and groups them according to these traits. On the basis of this diagram, the first two components explained 30.29% and 21.80%, respectively, and more than 52% of the variance of the total data, and the genotypes were divided into four categories. SC540 and KSC704 genotypes were included in the first group, SC500 and SC307 genotypes were included in the second group, KSC260 and KSC705 genotypes were included in the third group, and KSC700 and KSC706 genotypes were included in the fourth group (Fig. 10).

## Discussion

The analysis of variance (ANOVA) revealed significant differences among the maize genotypes for all agronomic traits evaluated, confirming genetic variability within the studied hybrids. Among the genotypes, KSC704 and KSC706 consistently exhibited superior performance across multiple physiological and biochemical traits, thereby establishing themselves as the most desirable genotypes. In contrast, SC540 and KSC260 were consistently ranked lowest, suggesting limited adaptability and inferior agronomic performance.

Correlation analysis demonstrated strong positive relationships between grain yield and key physiological traits such as chlorophyll a, chlorophyll b, phosphorus, carotenoids, and sodium, underscoring the importance of these parameters in enhancing yield. Proline also showed a positive correlation with seed protein, suggesting its involvement in stress tolerance and protein synthesis. However, antagonistic relationships were observed, including negative correlations between catalase and proline, as well as between seed protein and potassium, indicating potential trade-offs that should be considered when selecting genotypes for specific environments.

Principal Component Analysis (PCA) identified five major components that accounted for over 79% of the total variance. The first three components, heavily influenced by traits such as chlorophyll content, carotenoids, catalase activity, and grain yield, were particularly effective in explaining phenotypic diversity. These results highlight the crucial role of photosynthetic efficiency, oxidative stress mitigation, and nutrient assimilation in determining maize productivity under variable environmental conditions.

Genotypes such as KSC705, KSC400, and DC370 were frequently associated with favorable trait combinations in the PCA biplot and were grouped among the most desirable based on chlorophyll concentration and seed phosphorus content. Further validation through polygon (GGE biplot) analysis confirmed the superior performance of KSC705 and KSC400, as these genotypes were located farthest from the origin, indicating greater adaptability and trait stability. Conversely, SC540, situated near the origin, exhibited minimal responsiveness across environments and was deemed the least suitable genotype.

The ranking diagram based on the ideal genotype further reinforced the status of KSC705 and KSC400 as top-performing hybrids. Their consistent trait performance and environmental stability make them ideal candidates for breeding programs aimed at yield improvement and stress resilience. The integration of ANOVA, correlation matrices, PCA, and multivariate analyses enabled a robust and comprehensive evaluation framework that efficiently distinguished genotypes with both high productivity and stability. Similar graphical and statistical approaches have been successfully employed by researchers such as Shojaei et al.^10,14^, Nikzad Semeskandi et al.^15^, and Mousavi et al.^16^.

In conclusion, the findings of this study offer valuable insights for maize breeding strategies targeting enhanced yield and environmental adaptability. Future research should focus on elucidating the genetic mechanisms behind these trait associations and evaluating the field performance of the identified genotypes under a broader range of agro-ecological conditions to validate their potential for large-scale deployment.

## Conclusion

Maize hybrids demonstrate considerable genetic diversity, highlighting their potential for targeted genetic improvement. Among the evaluated genotypes, KSC705 and KSC706 consistently outperformed others across agronomic, physiological, and biochemical traits, establishing them as promising candidates for breeding programs. In contrast, SC540 and KSC260 exhibited poor adaptability and lower overall performance. Key physiological traits—including chlorophyll a, chlorophyll b, phosphorus, carotenoids, and sodium—showed strong positive correlations with grain yield, emphasizing the importance of photosynthetic efficiency and nutrient uptake. Principal Component Analysis (PCA) confirmed that these traits significantly contribute to genotype performance. Complementary multivariate analyses, such as polygon and ideal genotype ranking diagrams, further validated the stability and adaptability of genotypes KSC705 and KSC400 across diverse environments. Collectively, these findings offer a robust framework for identifying and selecting high-yielding, stable maize hybrids suitable for cultivation in a variety of agro-climatic conditions, and underscore the need for future large-scale field evaluations to confirm their practical potential.

## Data Availability

All data supporting the conclusions of this article are included in this article.
